# Accumulative occlusion time correlates with postoperative pulmonary complications in patients undergoing pelvic and sacrum tumor resection assisted by abdominal aortic balloon occlusion: a retrospective cohort study

**DOI:** 10.1186/s12891-020-03343-w

**Published:** 2020-05-16

**Authors:** Junjun Xu, Huiying Zhao, Xiaodan Zhang, Yi Feng

**Affiliations:** 1 Department of Anesthesiology, Peking University People’s Hospital, No. 11 Xizhimen South Street, Xicheng District, Beijing, 10044 China; 2 Department of Critical Care Medicine, Peking University People’s Hospital, No. 11 Xizhimen South Street, Beijing, China

**Keywords:** Abdominal aortic balloon, Pelvic and sacrum tumor resection, Postoperative pulmonary complications, Ischemia-reperfusion lung injury

## Abstract

**Background:**

Postoperative pulmonary complications (PPCs) seems to be high in patients undergoing pelvic and sacrum tumor resection assisted by abdominal aortic balloon occlusion. We hypothesized that the accumulative occlusion time (AOT) of the abdominal aortic balloon may be predictive of PPCs. The objective of the study was to identify the influence of AOT on PPCs.

**Methods:**

Retrospectively analyzed perioperative factors of 584 patients who underwent pelvic and sacrum tumor resection assisted by abdominal aortic balloon occlusion in our hospital from January 1, 2016 to December 31, 2018. PPCs including suspected pulmonary infection, atelectasis, pulmonary edema, pleural effusion, respiratory failure were clinically diagnosed. Perioperative parameters among patients with and without PPCs were compared. A receiver operating characteristic (ROC) analysis was conducted to evaluate the discriminative power of AOT with regard to PPCs. A multivariate logistic-regression model was finally established to identify independent risk factors for PPCs.

**Results:**

The incidence of PPCs was 15.6% (91 patients). The median AOT in PPCs group was significantly higher than that in non-PPCs group (*P* <  0.001). The hospital stay was significantly prolonged in PPCs group (*P* <  0.001). The ROC analysis showed an AOT of 119 min as the threshold value at which the joint sensitivity (88.60%) and specificity (31.87%) was maximal. Finally, AOT ≥ 119 min (*P* = 0.046; odds ratio (OR) = 2.074), age (*P <* 0.001; OR = 1.032), ASA grade III (*P* = 0.015; OR = 3.264), and estimated blood loss (*P* = 0.022; OR = 1.235) were independent risk factors of PPCs by multivariate logistic regression analysis.

**Conclusion:**

The incidence of PPCs in patients undergoing the pelvic and sacrum tumor surgery assisted by abdominal aortic balloon occlusion was 15.6%. AOT ≥ 119 min was an independent predictor for PPCs. Surgeons should strive to minimize the AOT within 2 h.

## Background

Pelvic and sacrum tumors have an insidious onset and are difficult to diagnose at an early stage; in fact, when the tumor is eventually diagnosed, it has grown large and usually invades adjacent blood vessels, nerves and the rectum. The surgical excision of pelvic and sacrum tumors is very challenging, and is often associated with massive bleeding and a longer duration of surgery. Aortic occlusion is a surgical technique to minimize ongoing hemorrhage by decreasing distal arterial blood flow and pressure to injured organs or vessels [1], which is classically achieved by cross-clamping the aorta. In the past few decades, there has been increasing use of endovascular balloon occlusion of the aorta in the management of traumatic hemorrhage [2] and other clinical settings, such as surgeries of pelvic and sacrum tumors [3–5], prophylactic use in women with abnormal placentation [6] and patients with non-traumatic out-of-hospital cardiac arrest [7]. The application of abdominal aortic balloon occlusion in the excision of pelvic and sacrum tumors can effectively control intraoperative bleeding, shorten the duration of surgery, reduce the surgical complications, and improve the effectiveness and safety of the surgery [3, 4].

Compared with other malignant tumors, primary malignant bone tumors usually develop in young adults which have better preoperative basal pulmonary function. However, in the clinical setting, we found that the incidence of postoperative pulmonary complications (PPCs) is high, which may be associated with the longer operation and mechanical ventilation time, as well as greater intraoperative blood loss and blood transfusion. Moreover, the ischemia-reperfusion lung injury following the deflation of the abdominal aortic balloon may also contribute to the PPCs. As the extent of tissue injury usually relates to the extent of reduction in blood flow and to the length of the ischemic period, which influence the cellular level of ATP production and the reduction of intracellular pH [8], we hypothesized that the accumulative occlusion time (AOT) of the abdominal aorta occlusion may be a predictive factor of PPCs in patients undergoing pelvic and sacrum tumor resection assisted by abdominal aorta occlusion.

Previous studies on lung injury after aortic balloon application have mainly been conducted in animals [9, 10], or have included a small sample size [11]. Due to the low incidence of pelvic and sacrum tumors, to our knowledge, no study has assessed the incidence of and factors associated with PPCs in patients undergoing pelvic and sacrum tumor resection with abdominal aortic balloon occlusion. In the present study, we aimed to retrospectively analyze the factors associated with PPCs in these patients, especially the influence of AOT on the PPCs, so as to recommend optimal perioperative management measures and improve patient prognosis.

## Methods

### Study design overview

It was a retrospective cohort study approved by our local institutional review board (IRB no.2019PHB038–01; approved on February 28, 2019). We reviewed the medical records of 584 consecutive patients who underwent pelvic and sacrum tumor resection assisted by abdominal aortic balloon occlusion in Peking University People’s Hospital from January 1, 2016 to December 31, 2018.

### Surgical technique

After induction of general anesthesia, a balloon dilation catheter (MAXI LD; Cordis, a Johnson and Johnson company, Bridgewater, New Jersey) was inserted into the lower abdominal aorta through an 11F percutaneous introducer sheath (CROSSOVER; Cordis) inserted into the femoral artery about 1 h before surgery. An appropriate location of the balloon was confirmed distal to the renal arteries and proximal to abdominal aortic bifurcation under fluoroscopy. When the tumor was fully exposed, the abdominal aortic balloon was inflated at an appropriate time according to the experience of the surgeon to facilitate the surgical operation and reduce bleeding. The occlusion time of the balloon was determined by the surgeon during the surgical procedure. To avoid a sudden decrease of circulating blood volume, the balloon was deflated gradually. The occlusion of the balloon may be applied more than once as necessary during the surgery. Massive infusion and vasopressors were often needed. The balloon catheter was removed after surgery. The accumulative occlusion time (AOT) of the abdominal aorta was recorded.

### Anesthesia and postoperative pain management

All patients were placed under general anesthesia. After induction with intravenous propofol, sufentanil and cis-atracurium, endotracheal intubation and mechanical ventilation were performed. Maintenance anesthesia consisted of continuous infusion of propofol, remifentanil and cis-atracurium. Cis-atracurium was stopped infusing at least 1 h before the end of surgery. All patients received routine monitoring of invasive arterial pressure, electrocardiogram, pulse oxygen saturation, end-tidal carbon dioxide, central venous pressure, bispectral index (BIS), nasal temperature, urine output and arterial blood gas. Two large bore peripheral intravenous catheters were placed. The fluid of choice for these procedures was lactated Ringer’s solution as crystalloid and 6% hydroxyethylstarch 130/0.4 as colloid. Blood transfusions, including transfusion of packed red blood cells (PRBCs) and plasma, were performed based on the amount of intraoperative bleeding, the level of hematocrit, and the anesthesiologists’ judgments. Postoperative analgesia was delivered through a patient-controlled analgesia pump.

### Diagnosis of postoperative pulmonary complications

In accordance with the previous studies [12, 13], postoperative pulmonary complications included suspected pulmonary infection, atelectasis, pulmonary edema, pleural effusion and respiratory failure. In this study, these diagnoses were identified in medical records, radiographic reports, and/or the discharge summary. The definitions of PPCs [12, 13] were listed in Table 1.

**Table 1:** Definitions of postoperative pulmonary complications

Complications	Definitions
Suspected pulmonary infection	General signs of infection, at least one of the following criteria: patient received antibiotics, core body temperature > 38 °C, leukocytosis > 12,000 cells per μL; and signs of an infection of pulmonary origin, at least one of the following criteria: new or changed sputum, or new or changed lung opacity on chest X-ray when clinically indicated.
Atelectasis	Lung opacification with shift of the mediastinum, hilum, or hemidiaphragm towards the affected area and compensatory overinflation of the adjacent non-atelectatic lung.
Pulmonary edema	Defined as diffuse alveolar interstitial infiltrates with dyspnea and rales related to left ventricular failure, confirmed by one of the following: echocardiography, pulmonary catheter, or clinical improvement with specific treatment.
Pleural effusion	Chest X-ray demonstrating blunting of the costophrenic angle, loss of the sharp silhouette of the ipsilateral hemidiaphragm in upright position, evidence of displacement of adjacent anatomical structures, or (in supine position) a hazy opacity in one hemithorax with preserved vascular shadows.
Respiratory failure	
Mild	PaO_2_ < 60 mmHg, 8 kPa, or SpO_2_ < 90% in room air but responding to mask or nasal supplementary oxygen (excluding hypoventilation).
Intermediate	PaO_2_ < 60 mmHg, 8 kPa, or SpO_2_ < 90% and needing invasive or non-invasive mechanical ventilation (excluding hypoventilation)
Severe	PaO_2_-to-FiO_2_ ratio < 300 mmHg or 40 kPa regardless of level of PEEP, needing invasive mechanical ventilation (acute lung injury or acute respiratory distress syndrome).

*Abbreviation*: *PaO*_*2*_ partial pressure of oxygen in arterial blood, *SpO*_*2*_ peripheral blood oxygen saturation, *FiO*_*2*_ fractional concentration of oxygen in inspired air; *PEEP* positive end-expiratory pressure

### Preoperative and intraoperative parameters and postoperative outcomes

Patients who had developed PPCs during their hospital stay were compared with their non-PPCs counterparts. Preoperative parameters included age, gender, ASA grade, body mass index (BMI), smoking status (current smoker), pulmonary co-morbidities (including lung cancer, lung metastases, chronic obstructive pulmonary disease, asthma, etc.), preoperative hemoglobin value and preoperative albumin value. The intraoperative parameters included duration of surgery, intraoperative estimated blood loss, AOT of abdominal aortic balloon.

The main outcome was the incidence of PPCs occurring during the hospital stay. According to the severity of PPCs, patients were classified as mild (PaO_2_ ≥ 60 mmHg, 8 kPa, or SpO_2_ ≥ 90% in room air and only needing mask or nasal supplementary oxygen), moderate (PaO_2_ < 60 mmHg, 8 kPa, or SpO_2_ < 90% in room air but responding to mask or nasal supplementary oxygen, excluding hypoventilation) and severe (PaO_2_ < 60 mmHg, 8 kPa, or SpO_2_ < 90% and needing invasive or non-invasive mechanical ventilation support) [12]. The secondary outcome was the length of hospital stay.

### Statistical data analyses

Stata software, version 15.1 (Stata Corp) was used to accomplish the following statistical analyses. Continuous data were expressed as means with standard deviations or as medians with interquartile ranges, depending on normality. Categorical variables were shown as proportions. In the univariate testing, patients who had developed PPCs were compared with their non-PPCs counterparts. For the comparison between the 2 groups, categorical variables were analyzed using Pearson chi-square tests or Fisher exact tests as appropriate. Continuous variables were performed using Kruskal-Wallis equality-of-populations rank tests. The receiver operating characteristic (ROC) analysis was conducted to evaluate the discriminative power of AOT with regard to PPCs. The area under curve (AUC) with its 95% confidence interval (CI) was then extrapolated. The best cutoff value of the AOT was identified according to the sensitivity and specificity. Predictors with a *P* value of < 0.1 on univariate analysis were identified to be risk factors of PPCs. The variance inflation factor (VIF) and tolerance were used to test the multicollinearity of the risk factors. A VIF > 10 or tolerance < 0.1 was identified to be significant multicollinearity. Binary multivariate logistic regression was used to determine independent risk factors of PPCs. The Hosmer-Lemeshow test was used to estimate the goodness of fit for the logistic regression mode. A coefplot was performed to plot the regression coefficients. We used a nomogram to demonstrate the risk points and probability of independent risk factors for PPCs. A *P* value of < 0.05 in 2-sided tests was statistically significant.

## Results

### Study population

Of 591 patients initially identified as potential candidates, 7 patients were excluded from the final analysis because the abdominal aortic balloon was placed in the abdominal aorta but not used during the surgery (*n* = 4); or the occlusion time of the abdominal aortic balloon was not recorded (*n* = 3). Therefore, a total of 584 patients were included in the study. The preoperative variables and the intraoperative variables were summarized (Table 2).

**Table 2:** Perioperative variables of patients with or without PPCs

Variables	Overall (*n* = 584)	With PPCs (*n* = 91)	Without PPCs (*n* = 493)	*P* value
Age (y)	45.5 (31–55)	51 (36–62)	44 (30–54)	< 0.001
Gender, male (%)	307 (52.6%)	43 (47.3%)	264 (53.6%)	0.269
BMI	23.0 (20.3–25.3)	23.0 (20.0–25.7)	22.9 (20.4–25.1)	0.775
ASA grade				0.001
Grade I (%)	158 (27.1%)	15 (16.5%)	143 (29.0%)	
Grade II (%)	388 (66.4%)	63 (69.2%)	325 (65.9%)	
Grade III (%)	38 (6.5%)	13 (14.3%)	25 (5.1%)	
Pulmonary co-morbidities (%)	63 (10.8%)	12 (13.2%)	51 (10.3%)	0.422
Current smoker (%)	64 (11.0%)	14 (15.4%)	50 (10.1%)	0.141
Preoperative Albumin (g/L)	41.3 (38.8–44)	40.7 (37.1–43.3)	41.4 (39.0–44.1)	0.066
Preoperative Hemoglobin (g/L)	130 (118–142)	128 (113–142)	130 (119–142)	0.307
Estimated blood loss (L)	1.5 (0.8–2.5)	2.15 (1.0–3.9)	1.5 (0.8–2.2)	< 0.001
Duration of surgery (h)	4.1 (3.2–5.6)	5.2 (3.5–7.1)	4.0 (3.1–5.3)	< 0.001
Accumulative occlusion time (min) occlusion time (min)	80 (58–104.5)	91 (66–129)	77 (57–102)	< 0.001
≥ 119 min (%)	85 (14.6%)	29 (31.9%)	56 (11.4%)	< 0.001
Length of stay (d)	22 (18–28)	27 (20–36)	22 (18–27)	< 0.001

The data are presented as the median (25–75% interquartile range) or number (percentage)

*Abbreviation*: *PPCs* postoperative pulmonary complications, *BMI* body mass index, *ASA* American Society of Anesthesiologists

### Summary of postoperative pulmonary complications and the length of hospital stay

Of the 584 patients, 91 (15.6%) exhibited pulmonary complications, including suspected pulmonary infection in 64 (11.0%, with 60 patients diagnosed with Chest X-ray, 4 patients diagnosed based on clinical symptoms and signs, including fever, leukocytosis and new sputum), pleural effusion in 36 (6.2%, with all the patients diagnosed with Chest X-ray), atelectasis in 17 (2.9%, with 12 patients diagnosed with CT scans, 5 patients diagnosed with Chest X-ray as recorded in the radiographic reports), pulmonary edema in 6 (1.0%, with 5 patients diagnosed with Chest X-ray, 1 patient diagnosed with Chest X-ray and CT scans) and respiratory failure in 4 (0.7%, with all the patients diagnosed with arterial blood gas analysis).

Of the 91 patients with PPCs, according to the severity of PPCs, 87 patients were classified as mild (95.6%), 2 patients were classified as moderate (2.2%), and 2 patients were classified as severe (2.2%). No death was noted in any of the cases.

Compared with non-PPCs group, the length of hospital stay was significantly longer in PPCs group (*P* <  0.001) (Table 2).

### ROC analysis on the prediction of accumulative occlusion time for PPCs

**Fig. 1:**
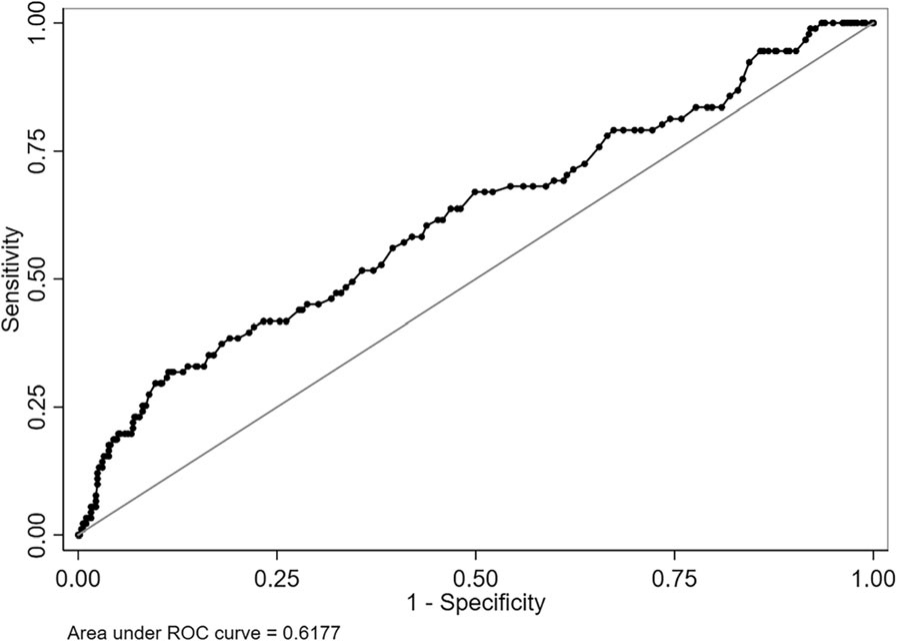
The ROC curve of accumulative occlusion time for the prediction of postoperative pulmonary complications

The ROC analysis of AOT showed an AUC 0.62 (95% CI = 0.55–0.68) for the prediction of PPCs (see Fig. 1). According to the ROC curve, an AOT of 119 min was found to be the optimal cutoff value with the maximum joint sensitivity (88.60%) and specificity (31.87%). Therefore, AOT ≥ 119 min was determined as the threshold value for predicting the risk of PPCs. On the basis of the threshold value of AOT, there were 499 patients with AOT < 119 min and 85 patients with AOT ≥ 119 min, respectively, which were also summarized in Table 2.

### Univariate analysis

The AOT of abdominal aortic balloon (*P* <  0.001) was significantly longer in PPCs group, and the proportion of AOT equal or longer than 119 min (*P* <  0.001) was also higher in PPCs group when compared with non-PPCs group. Patients with PPCs were older (*P* <  0.001), had a higher proportion of ASA grade III and a lower proportion of ASA grade I. The preoperative albumin value showed a trend toward a higher risk of PPCs (*P* = 0.066). Patients with PPCs had a significantly higher intraoperative estimated blood loss volume (*P* <  0.001) and a longer duration of surgery (*P* <  0.001) when compared with non-PPCs patients. No significant difference in gender, BMI, current smoker, pulmonary co-morbidities, and preoperative hemoglobin value was observed.

### Multivariate analysis

**Fig. 2:**
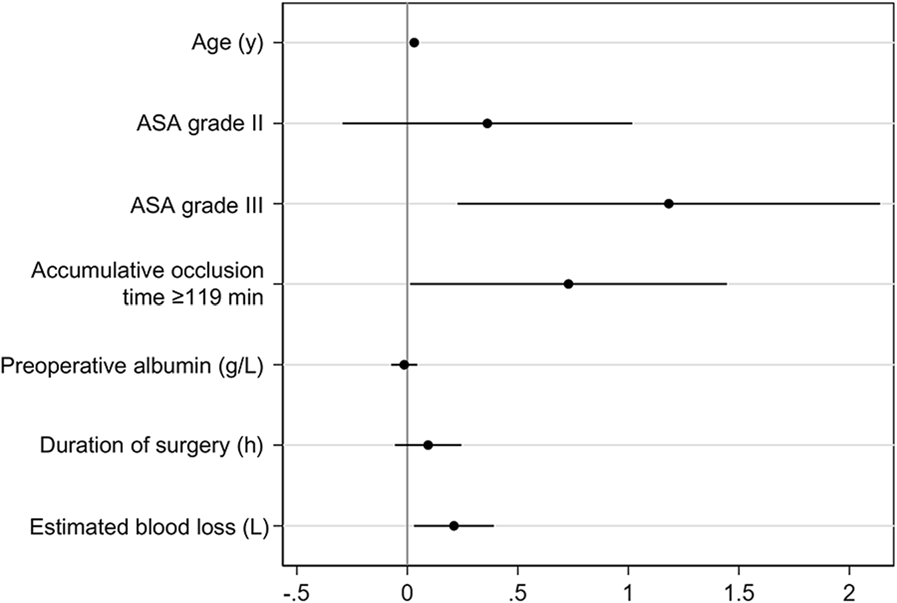
Coefplot of the Logistic regression coefficient

We tested the multicollinearity of all the variables with a *P* value of < 0.1 on univariate analysis as mentioned above (Table 3). The results showed that the largest VIF of the above variables was 1.93 and the smallest tolerance was 0.518. Therefore, we included all of them in the binary multivariate logistic regression model, and we found that AOT ≥ 119 min, age, ASA grade III, and estimated blood loss were independent risk factors of PPCs in patients undergoing excision of pelvic and sacrum tumors assisted by abdominal aortic balloon (Table 4). The Hosmer-Lemeshow test showed the fit for the multivariate logistic regression model was good (*P* = 0.165, chi2 = 602.9). The coefplot of the regression coefficients was shown in Fig. 2. These results revealed that AOT ≥ 119 min was an independent risk factor of PPCs after adjusting the influence of age, ASA grade, preoperative albumin value, estimated blood loss volume, and duration of surgery.

**Table 3:** Multicollinearity test of risk factors of PPCs

Risk factors	VIF	Tolerance
Age (y)	1.16	0.859
ASA grade	1.16	0.861
Preoperative albumin (g/L)	1.06	0.946
Duration of surgery (h)	1.93	0.518
Estimated blood loss (L)	1.85	0.542
Accumulative occlusion time (min)	1.57	0.637

*Abbreviation*: *PPCs* postoperative pulmonary complications, *ASA* American Society of Anesthesiologists, *VIF* variance inflation factor

**Table 4:** Multivariate logistic regression analysis of PPCs and clinical variables

Variables	OR	*P* value	95% CI
Accumulative occlusion time ≥ 119 min (%)	2.074	0.046	1.013–4.244
Age (y)	1.032	< 0.001	1.014–1.050
ASA Grade III (%)	3.264	0.015	1.255–8.487
Estimated blood loss (L)	1.235	0.022	1.031–1.479

*Abbreviation*: *PPCs* postoperative pulmonary complications, *ASA* American Society of Anesthesiologists

### Nomogram

**Fig. 3:**
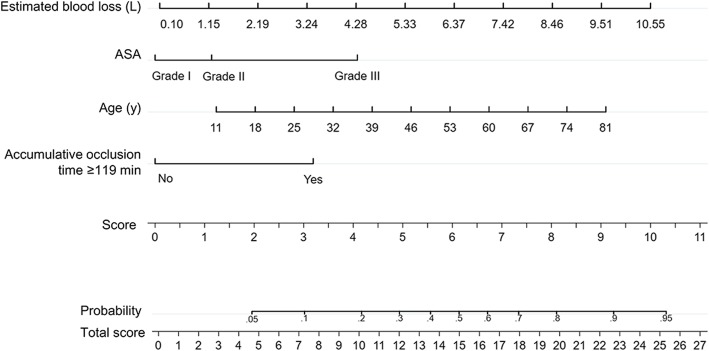
Nomogram for postoperative pulmonary complications using the independent risk factors

Through the multivariate logistic regression model, we built a prognostic nomogram incorporating the above independent prognostic factors for visualization and facilitating clinical practice as shown in Fig. 3.

## Discussion

To our knowledge, this is the first study to assess the risk factors associated with PPCs in patients undergoing pelvic and sacrum tumor resection assisted by abdominal aortic balloon occlusion, especially the influence of AOT on the incidence of PPCs in these patients. A retrospective analysis of 584 patients showed that the incidence of PPCs was 15.6%, with suspected pulmonary infection, pleural effusion and atelectasis as the most common complications. The ROC analysis showed an AOT of 119 min as the optimal cutoff value with regard to risk of PPCs. Finally, an AOT ≥ 119 min was identified to be an independent risk factor of PPCs after adjusting the influence of age, ASA grade, preoperative albumin value, estimated blood loss, and duration of surgery.

PPCs after major surgery are an important cause of death and morbidity [13, 14], and the duration of hospital stay was found to be significantly longer in patients at high risk of PPCs than in low-risk patients [15]. Therefore, the early identification of patients at high risk of PPCs can help us initiate preventive measures and provide appropriate treatment, which has important clinical significance. Recently, a multicenter prospective observational study named POPULAR showed that the incidence of PPCs in patients who underwent non-cardiac surgery with general anesthesia and muscle relaxants was 7.6%, and that the high-risk types of surgery included intrathoracic surgery and upper abdominal laparotomy [12]. In our study, the incidence of PPCs in patients undergoing pelvic and sacrum tumor resection assisted by abdominal aortic balloon occlusion is 15.6%, which is higher than the overall incidence of PPCs in the POPULAR study. Although most of the patients with PPCs were classified as mild, patients with PPCs have longer hospital stays compared with non-PPCs patients, which is consistent with previous result [15]. As predictive factors for PPCs, age [12, 13, 15], ASA physical status [12, 13, 15] and estimated blood loss [16] have already been discussed thoroughly. For the first time, we found that patients with longer AOT had a higher probability of PPCs in these patients.

Pelvic and sacrum tumors are rich in blood supply, and hence, surgeries are often accompanied by extensive bleeding that can be difficult to control during tumor resection. In severe cases, extensive hemorrhage may lead to hemorrhagic shock or even death. The application of abdominal aortic balloon occlusion enables surgeons to obtain a good surgical field and surgical conditions, control the amount of bleeding, shorten the duration of surgery, and improve the thoroughness and safety of pelvic and sacrum tumor surgery [3, 4]. However, accompanied by the inflation and deflation of the abdominal aortic balloon, the blood supply of the distal tissue is interrupted and recovered, and then ischemia-reperfusion (I/R) injury occurs. In addition to the damage that occurs in the tissue which was subjected to the ischemia and reperfusion process, distal organs are also affected by this process, which may result in multiple organ dysfunction [17, 18], including lung injury [11, 19]. An animal study has assessed the occurrence of ischemia-reperfusion injury in cases undergoing abdominal aortic balloon occlusion for 30 min, 60 min and 120 min [9], and showed that the peak inspiratory pressure was significantly greater in samples with balloon occlusion of 120 min compared with baseline. Moreover, lung tissue biopsy of the 3 groups all showed alveolar congestion and atelectasis. So, ischemia-reperfusion lung injury induced by applying abdominal aortic balloon occlusion could contribute to the lung injury in these patients and the extent of the lung injury may be occlusion time-dependent. This may be part of the reason why the incidence of PPCs in our study is so high. Clinicians should note that abdominal aorta balloon is a good tool for reducing bleeding, but the risk of lung injury should not be ignored.

The appropriate time to block the abdominal aortic balloon remains unclear. Current studies generally recommend limiting the single continuous balloon blockade time to 60–90 min to avoid damage to the arterial wall, ischemic necrosis of the distal limb, organ damage or multiple organ dysfunctions, and the balloon can be inflated again after 10–15 min if needed [20–22]. To our knowledge, no study has assessed the effect of the AOT when the aortic balloon was occluded more than once. In our study, we found that the AOT ≥ 119 min was an independent risk factor for PPCs, and patients with AOT ≥ 119 min were 2.07 times more likely to develop such complications compared to those with AOT less than 119 min. This result is of important clinical significance. It means that surgeons shouldn’t only consider the occlusion time of a single time, but also the AOT when the occlusion was conducted more than once. Minimizing the AOT less than 2 h could be set as a first goal. However, the weight of AOT ≥ 119 min on the prediction of PPCs is not very great in the nomogram of this study.

The development of ischemia-reperfusion injury associated with sustained aorta balloon occlusion has prompted surgeons to seek measures to improve the blood supply of the distal tissues while maintain the effectiveness of controlling hemorrhage. In recent years, interests in intermittent [23] and partial blockade [24] are increasing, which involves the intermittent inflation to ensure tissue perfusion or partial inflation of the balloon to allow a certain degree of blood flow; however, none of these methods have been verified in large clinical trials. A new technique called endovascular variable aortic control (EVAC) has emerged as an promising alternative to partial resuscitative endovascular balloon occlusion of the aorta (REBOA), in which automated control of aortic occlusion is used to precisely and dynamically regulate distal aortic flow across the full spectrum from complete occlusion to full unimpeded flow [25]. Compared with standard REBOA, EVAC can improve hemodynamics, decrease metabolic derangements and resuscitation requirements over a 45 min intervention duration [26], but it still needs verification whether it is suitable for pelvic and sacrum tumor resections.

As to reduce the lung injury following ischemia-reperfusion caused by abdominal aorta balloon occlusion, recent studies have given three clues. First, previous studies have showed that reactive oxygen species (ROS) and the inflammatory cytokines play an important role in the pathogenesis of ischemia-reperfusion induced lung injury [27, 28], and several protective agents have been introduced to target these. In a hind limb I/R model in rats, edaravone exert a potent protective effect against lung injury induced through its antioxidant and anti-inflammatory activities [29]. Another animal study has also shown that Lazaroid U-74389G (an anti-lipid peroxidation drug) can reduce lung ischemia-reperfusion injury once the thoracoabdominal aortic occlusion has been relieved [10], and remains an avenue of future research. Second, in a rat model, the remote postconditioning was able to minimize the inflammatory lesion of the lung parenchyma of rats undergoing ischemia and reperfusion process caused by clamping the abdominal aorta [30], which seems more practical in clinical setting than remote-preconditioning. Third, a study showed that early ventilatory intervention will block progression to acute respiratory distress syndrome (ARDS) induced by ischemia-reperfusion and peritoneal sepsis. Airway pressure release ventilation (APRV) maintains a sustained airway pressure over a large proportion of the respiratory cycle, and therefore this ventilation strategy has a high pressure-time profile which can prevent alveolar edema and maintain alveolar stability throughout the ventilatory cycle [31]. In conclusion, comprehensive measures are needed to prevent the lung injury following ischemia-reperfusion caused by abdominal aorta balloon occlusion.

The present study has certain limitations. First, due to the limitations of retrospective studies, the collection of clinical data remains imperfect. For example, “smoking status” in patient data refers to current smokers who have not quit smoking, and no distinction was made between patients who had never smoked and those who had quit smoking. Second, the diagnosis of PPCs depends on the clinician’s judgment, and was then confirmed via imaging and laboratory examinations. Therefore, some diseases without apparent symptoms might be ignored, such as mild pleural effusion, and atelectasis, which may lead to the underestimation of the incidence of postoperative pulmonary complications. Finally, the sample size of this study was relatively small, which limits the applicability of the results. In the future, a prospective study with a large sample size can be conducted, with the comprehensive collection of clinical data. In such studies, the factors associated with PPCs and their impact on short-term mortality and long-term survival rate can be assessed, and a series of measures can be initiated to reduce ischemia-reperfusion lung injury and provide suitable interventions. In addition, the present study could only observe a certain relationship between longer occlusion time and pulmonary complications, but their causal relationship was not clear, which may be further verified in the future research.

## Conclusions

In conclusion, the incidence of PPCs in patients undergoing the pelvic and sacrum tumor surgery assisted by abdominal aortic balloon occlusion was 15.6%. AOT ≥ 119 min was an independent risk factor for PPCs after adjusting the influence of age, ASA grade, preoperative albumin value, estimated blood loss, and duration of surgery. Clinicians should note that abdominal aorta balloon is a good tool for reducing bleeding, but the risk of lung injury should not be ignored. Surgeons should strive to minimize the AOT within 2 h.

## Data Availability

The datasets used and analyzed during the current study are available from the corresponding author on request.

## Abbreviations

AOT: Accumulative occlusion time
APRV: Airway pressure release ventilation
ARDS: Acute respiratory distress syndrome
AUC: Area under curve
BIS: Bispectral index
BMI: Body mass index
CI: Confidence interval
EVAC: Endovascular variable aortic control
FiO_2_: Fractional concentration of oxygen in inspired air
I/R: Ischemia-reperfusion
OR: Odds ratio
PaO_2_: Partial pressure of oxygen in arterial blood
PEEP: Positive end-expiratory pressure
PPCs: Postoperative pulmonary complications
PRBCs: Packed red blood cells
REBOA: Resuscitative endovascular balloon occlusion of the aorta
ROC: Receiver operating characteristic
ROS: Reactive oxygen species
SpO_2_: Peripheral blood oxygen saturation
VIF: Variance inflation factor
